# Metabolomics Integration in Assisted Reproductive Technologies for Enhanced Embryo Selection beyond Morphokinetic Analysis

**DOI:** 10.3390/ijms25010491

**Published:** 2023-12-29

**Authors:** Soraia Pinto, Bárbara Guerra-Carvalho, Luís Crisóstomo, António Rocha, Alberto Barros, Marco G. Alves, Pedro F. Oliveira

**Affiliations:** 1 Centre for Reproductive Genetics Alberto Barros, 4100-012 Porto, Portugal; soraiapinto.pt@gmail.com (S.P.); abarros@med.up.pt (A.B.); 2 LAQV-REQUIMTE, Department of Chemistry, University of Aveiro, 3810-193 Aveiro, Portugal; barbaraggcarvalho@gmail.com; 3 Institute of Biomedicine, University of Turku, 20014 Turku, Finland; luis.crisostomo@utu.fi; 4 CECA/ICETA–Centro de Estudos de Ciência Animal, Institute of Biomedical Sciences Abel Salazar (ICBAS), University of Porto, 4200-135 Porto, Portugal; amrocha@icbas.up.pt; 5 i3S—Instituto de Investigação e Inovação em Saúde, University of Porto, 4200-135 Porto, Portugal; 6 Department of Pathology, Faculty of Medicine, University of Porto, 4200-319 Porto, Portugal; 7 Institute of Biomedicine (iBiMED), Department of Medical Sciences, University of Aveiro, 3810-193 Aveiro, Portugal; alvesmarc@gmail.com

**Keywords:** embryo quality evaluation, morphokinetic assessment, embryo metabolomics, pyruvate, glutamine

## Abstract

Embryo quality evaluation during in vitro development is a crucial factor for the success of assisted reproductive technologies (ARTs). However, the subjectivity inherent in the morphological evaluation by embryologists can introduce inconsistencies that impact the optimal embryo choice for transfer. To provide a more comprehensive evaluation of embryo quality, we undertook the integration of embryo metabolomics alongside standardized morphokinetic classification. The culture medium of 55 embryos (derived from 21 couples undergoing ICSI) was collected at two timepoints (days 3 and 5). Samples were split into Good (n = 29), Lagging (n = 19), and Bad (n = 10) according to embryo morphokinetic evaluation. Embryo metabolic performance was assessed by monitoring the variation in specific metabolites (pyruvate, lactate, alanine, glutamine, acetate, formate) using ^1^H-NMR. Adjusted metabolite differentials were observed during the first 3 days of culture and found to be discriminative of embryo quality at the end of day 5. Pyruvate, alanine, glutamine, and acetate were major contributors to this discrimination. Good and Lagging embryos were found to export and accumulate pyruvate and glutamine in the first 3 days of culture, while Bad embryos consumed them. This suggests that Bad embryos have less active metabolic activity than Good and Lagging embryos, and these two metabolites are putative biomarkers for embryo quality. This study provides a more comprehensive evaluation of embryo quality and can lead to improvements in ARTs by enabling the selection of the best embryos. By combining morphological assessment and metabolomics, the selection of high-quality embryos with the potential to result in successful pregnancies may become more accurate and consistent.

## 1. Introduction

In recent years, the incidence of infertility has been increasing, which can be attributed to various social, biological, and biochemical factors. The World Health Organization (WHO) defines infertility as the inability of a couple to achieve pregnancy after one year of sexual intercourse without contraception [1]. It is estimated that infertility affects between 4 and 14% of the population globally, making it a significant public health burden. Assisted reproductive technologies (ARTs) have emerged as a solution to this problem, with the WHO advocating for increased accessibility to such treatments. However, success rates of ARTs are still below expectations, indicating a need for further investigation into the biochemical processes involved in ART techniques, particularly during embryonic development, and the identification of biomarkers associated with the outcome of ART programs [2]. One of the significant challenges of ARTs is the optimization of embryo selection. The existing approaches to gauging the potential for embryo implantation primarily hinge on morphological assessment. However, this method does not consistently provide accurate predictions of successful implantation [3].

Metabolomics, a quantitative and non-invasive technology, has recently emerged as a promising tool for measuring metabolites present in cells, tissues, body fluids, and culture media. By quantifying the metabolites secreted by gametes and embryos during culture, metabolomics can provide insights into embryonic viability and help to identify embryonic biomarkers that may improve the outcomes of ART treatments [4,5]. Several studies have already established correlations between embryonic viability and carbohydrate, pyruvate, and amino acid metabolism during embryonic development [5,6,7,8,9,10]. Indeed, the metabolic performance of embryos post-compaction has emerged as a noteworthy biomarker indicative of superior-quality blastocysts [9,11]. Despite these promising findings, the lack of consensus in this field largely stems from the absence of standardization in the molecular assessment of embryo quality [12]. Therefore, this study aims to integrate metabolomics with the standardized morphological classification of embryos to identify biomarkers that can optimize the selection of the best embryo for transfer. By combining metabolomic and standardized morphological embryo evaluations, this study can provide valuable insights into the complex metabolic processes underlying embryonic development and quality. Ultimately, the integration of metabolomics in ARTs can offer a powerful tool for improving treatment outcomes and identifying biomarkers associated with embryonic viability and quality. The identification of such biomarkers can greatly enhance the efficiency and effectiveness of ART, leading to better pregnancy rates and improved clinical outcomes for infertile couples.

## 2. Results

### 2.1. Clinical Data and Embryo Characterization

As shown in Table 1, 21 intracytoplasmic sperm injection (ICSI) cycles were included in this study. All the treatments were made in accordance with clinical indication, and fertilization was achieved with the ICSI technique. The embryo culture was extended until day 5 (D5). The cycle characteristics are presented in Table 1. In 7 cycles, the treatment involved Preimplantation Genetic Testing (PGT) without fresh embryo transfer. In this type of procedure, the embryos that develop into blastocysts with good quality are biopsied and then cryopreserved for deferred transfer after genetic results. Of the remaining 14 cycles, 5 were performed with oocyte donation and in 9 cycles, the ICSI protocol has no additional technique. Apart from the PGT cycles, a fresh embryo transfer had to be called off during a particular treatment due to elevated progesterone serum levels. Consequently, in this scenario, embryos of good quality were preserved through vitrification for a subsequent transfer. Across all 13 remaining cycles, the fresh embryo transfers were successfully carried out, aligning with the precise number of embryos transferred in accordance with the established guidelines of the Centro de Genética Prof. Alberto Barros (CGR) clinic. The surplus good-quality embryos were vitrified for later use. A total of 110 culture medium samples from 55 oocytes were collected during the study period, 55 samples of culture medium from embryos on day 3 (D3) and 55 samples of culture medium from embryos on D5. Degenerated embryos, non-cleavage embryos, abnormal fertilization, and parthenogenetic embryos were excluded from statistical analyses.

The embryos were classified as described below. Based on morphological assessment on D5, a blastocyst of good quality was considered as BL3BB or better (BL5—AA, AB, BA, BB; BL4—AA, AB, BA, BB; BL3—AA, AB, BA, BB), and a Bad embryo was considered if it had inner cell mass or/and trophectoderm grade C (BL5/BL4/BL3—AC, CA, CC). An embryo was classified as Lagging if the embryo development was delayed considering the standardized timing of embryo observations established in the Istanbul Consensus [13]. From the 55 observed embryos, 26 were classified as Good, 19 as Lagging, and 10 as Bad embryos.

### 2.2. Embryo Quality at Day 5 Correlates with Metabolite Consumption or Release

**Figure 1:**
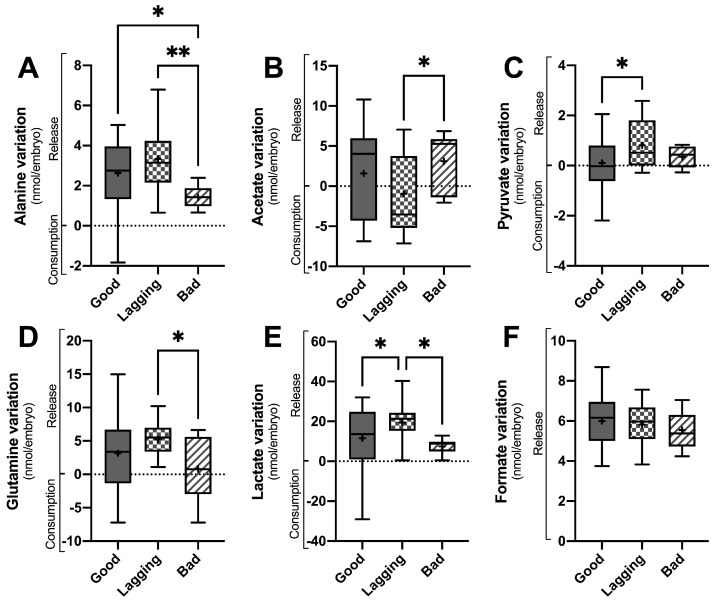
Metabolite variation in culture media of human embryos between day 3 (D3) and day 0 grouped according to their quality at day 5 (D5) (Good vs. Lagging vs. Bad). The figure shows the variation (Consumption or Release) in selected metabolites (Panel (**A**)-alanine; Panel (**B**)-acetate; Panel (**C**)-pyruvate; Panel (**D**)-glutamine; Panel (**E**)-lactate; Panel (**F**)-formate) quantified in the embryo culture media at the end of D3 (n = 55). Data are presented as mean ± standard deviation (S.D.). Normality and heteroscedasticity tests were performed, and univariate ANOVA was conducted on the selected metabolites identified in the embryo culture medium. Pairwise comparisons between groups were corrected for multiple hypotheses testing by Tukey’s post hoc test. All *p* values < 0.05 were considered statistically significant (* *p* < 0.05; ** *p* < 0.005). + represents group average.

**Figure 2:**
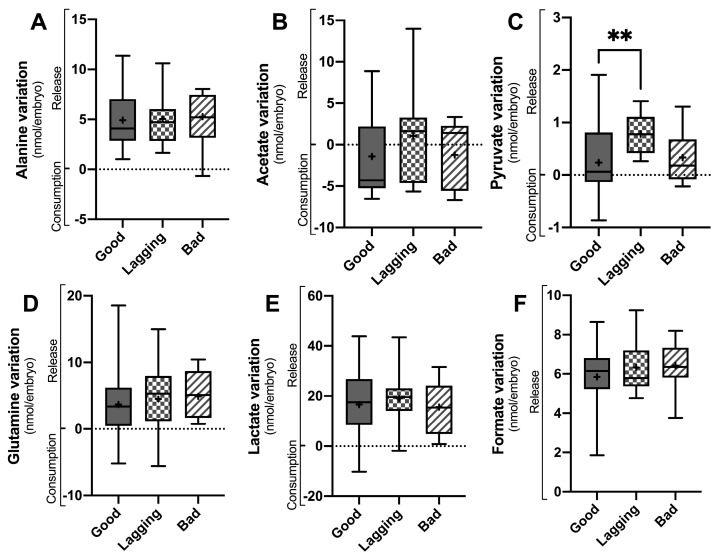
Metabolite variation in culture media of human embryos between day 3 (D3) and day 5 (D5) grouped according to their quality at D5 (Good vs. Lagging vs. Bad). The figure shows the variation (Consumption or Release) in selected metabolites (Panel (**A**)-alanine; Panel (**B**)-acetate; Panel (**C**)-pyruvate; Panel (**D**)-glutamine; Panel (**E**)-lactate; Panel (**F**)-formate) quantified in the embryo culture media at the end of D5 (n = 55). Data are presented as mean ± standard deviation (S.D.). Normality and heteroscedasticity tests were performed, and univariate ANOVA was conducted on the selected metabolites identified in the embryo culture medium. Pairwise comparisons between groups were corrected for multiple hypotheses testing by Tukey’s post hoc test. All *p* values < 0.05 were considered statistically significant (** *p* < 0.005). + represents group average.

Metabolite consumption and release by the embryos were assessed by ^1^H-NMR analysis of the embryo culture media on D3 (Figure 1 and Table 2) and on D5 (Figure 2 and Table 2). Alanine (Figure 1A and Figure 2A), acetate (Figure 1B and Figure 2B), pyruvate (Figure 1C and Figure 2C), glutamine (Figure 1D and Figure 2D), lactate (Figure 1E and Figure 2E), and formate (Figure 1F and Figure 2F) were the most relevant metabolites identified in the culture media of both D3 and D5. Embryos that were categorized into Good, Lagging, or Bad according to their quality at D5 were evaluated for metabolite consumption or production using ^1^H-Nuclear Magnetic Resonance (NMR) spectroscopy, and differences were analyzed by one-way ANOVA, followed by Tukey’s post hoc test.

On D3 of embryo culture, there were significant differences in the levels of alanine detected in the culture media. Specifically, both Good and Lagging embryos showed a higher release of alanine (2.63 ± 1.68 nmol/embryo and 3.34 ± 1.65 nmol/embryo, respectively) compared to Bad embryos (1.42 ± 0.57 nmol/embryo). Similarly, glutamine release was significantly higher in Lagging embryos (5.24 ± 2.69 nmol/embryo) than in Bad embryos (0.80 ± 4.66 nmol/embryo). In Figure 1D, it can be observed that the release of glutamine to the culture media by Good embryos was intermediate, with a mean value of 3.16 ± 4.94 nmol/embryo, compared to the values observed for Bad and Lagging embryos.

Lagging embryos consumed acetate (−0.94 ± 4.98 nmol/embryo), contrarily to Good (1.59 ± 5.23 nmol/embryo) and Bad (3.11 ± 3.66 nmol/embryo) embryos, which released it to the media (Figure 1B). Pyruvate (Figure 1C) and lactate (Figure 1E) release was higher in Lagging (0.66 ± 0.99 and 19.44 ± 9.05 nmol/embryo) embryos than in Good (0.10 ± 1.23 and 11.55 ± 15.05 nmol/embryo) and Bad (0.35 ± 0.42 and 7.49 ± 3.99 nmol/embryo) embryos. No differences were observed regarding formate release between Good (6.00 ± 1.18 nmol/embryo), Lagging (5.82 ± 1.06 nmol/embryo), and Bad (5.54 ± 0.94 nmol/embryo) embryos (Figure 1F).

Concerning D5 variation in metabolites (Figure 2), only pyruvate levels were significantly different between groups. Good embryos (0.23 ± 0.67 nmol/embryo, *p* = 0.0112) released significantly less pyruvate than Lagging embryos (0.78 ± 0.38 nmol/embryo), whereas Bad (0.33 ± 0.57 nmol/embryo) embryos’ pyruvate release was intermediate (Figure 2C). No differences were found concerning alanine (4.92 ± 2.83; 5.05 ± 2.62 and 5.27 ± 2.70 nmol/embryo) (Figure 2A), glutamine (3.10 ± 4.47; 4.48 ± 5.65 and 4.90 ± 3.42 nmol/embryo) (Figure 2D), lactate (16.60 ± 13.27; 19.03 ± 10.39 and 15.55 ± 10.99 nmol/embryo) (Figure 2E), and formate (5.85 ± 1.51; 6.33 ± 1.21 and 6.43 ± 1.23 nmol/embryo) release (Figure 2F) levels between Good, Lagging, and Bad embryos, respectively. Although no statistical difference was observed, Good (−1.43 ± 4.44 nmol/embryo) and Bad (−1.24 ± 4.16 nmol/embryo) embryos tended to consume acetate, whereas Lagging embryos (1.04 ± 5.78 nmol/embryo) tended to release it to the media (Figure 2B).

### 2.3. Metabolite Variation during the First 3 Days of Development Is a Strong Predictor of Embryo Quality at Day 5

Metabolite consumption or release by embryos was assessed in embryo culture medium on the first 3 days (D0–3) and from D3 to D5 (D3–5). Metabolite variations were compared between groups of embryos categorized according to their quality at D5 using MANCOVA, after adjusting for covariates. One of the assumptions to conduct MANCOVA was not met when analyzing the D0–3 period: The homogeneity of covariance matrices across groups is not assumed (Box’s M = 120.66, F (56, 2017.579) = 1.584, *p* = 0.004), but it is assumed in the D3–5 interval. Also, the homogeneity of variance of all dependent variables was confirmed with Levene’s test in both intervals. Linearity and multicollinearity were graphically assessed and were considered acceptable in both intervals. Therefore, we considered that the MANCOVA model was sufficiently robust.

In addition to the mathematical evaluation of the methodology, we have conducted a thorough deliberation while selecting the covariates to be integrated into the model. Our choices have been driven by their intrinsic physiological significance and relevance. Recent studies have established a direct proportion between the age of the donor of the female gamete and embryo oxygen consumption rate (OCR) during the preimplantation phase [14,15,16]. Although OCR and maximum OCR are crucial and measurable markers of early embryo metabolism and development, other factors such as DNA integrity, antioxidant pool, and pre-collection hormonal levels further contribute to embryo development [17,18,19]. The same variables act on the male gamete and contribute to the fate of the embryo during early development [20,21]. Age and body mass index (BMI) have been reported to be correlated with metabolic, antioxidant, and DNA integrity in both female and male gametes, with direct influence over early embryo development [18,19,20,21,22,23]. Therefore, we selected the broader and easily measurable “age” and “BMI” of both male and female as covariates in our MANCOVA model.

**Figure 3:**
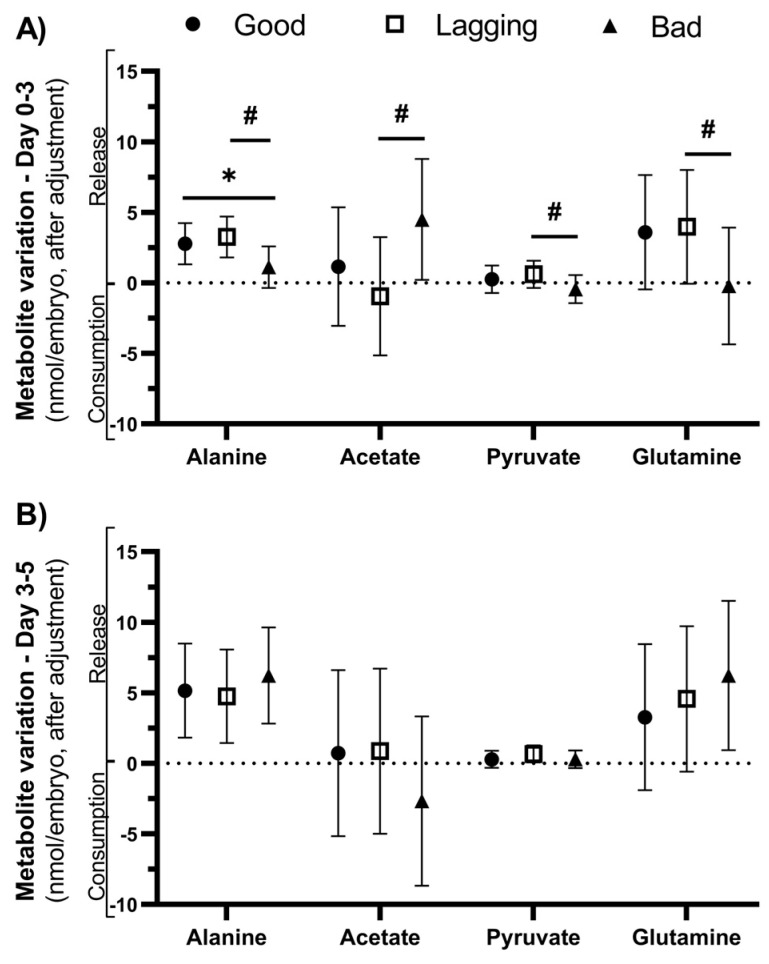
Metabolite variation observed on the first 3 days (D0–3) and from day 3 to day 5 (D3–5) in culture media of human embryos grouped according to their quality (Good vs. Lagging vs. Bad). The figure shows the metabolite differentials of relevant metabolites (alanine, acetate, pyruvate, and glutamine) observed on the first 3 days (Panel (**A**)) and from day 3 to day 5 (Panel (**B**)) (n = 55). Data are presented as mean ± standard deviation (S.D.). The impact of each metabolite differential on embryo quality at day 5 was assessed using a univariate F-test, after adjusting for the covariates. Pairwise comparisons between groups were corrected for multiple hypotheses testing by Šidak’s method. All *p* values < 0.05 were considered statistically significant (∗ vs. Good embryos; # vs. Lagging embryos).

In the D0–3 interval, metabolite differentials are discriminative of embryo quality category at D5 (Roy’s Largest Root = 0.644, F (7, 41) = 3.77, *p* = 0.003, partial η^2^ = 0.392), after controlling for covariates. The discriminant power of the model is supported by the differences in alanine (F (2, 46) = 6.428; *p* = 0.003), acetate (F (2, 46) = 4.940; *p* = 0.011), pyruvate (F (2, 46) = 3.443; *p* = 0.040), and glutamine (F (2, 46) = 3.448; *p* = 0.040). Pairwise comparisons were performed to identify which groups had different metabolite yields after incubation, corrected by Šidak’s method (Figure 3A).

After adjusting for confounding factors, alanine release during D0–3 was lower in Bad embryos (1.11 ± 1.48 nmol/embryo) than in Lagging embryos (3.25 ± 1.44 nmol/embryo, *p* = 0.003) and Good embryos (2.78 ± 1.45 nmol/embryo, *p* = 0.018). The remaining observed differences—acetate, pyruvate, and glutamine—were found comparing D0–3 differentials between Lagging and Bad embryos (Figure 3A and Table 3). Interestingly, Lagging embryos display a consumption of acetate contrary to the other groups, which released it (Figure 3A). Bad embryos consumed pyruvate and glutamine, energetic substrates, contrary to the other groups. However, pyruvate differentials vary within limited ranges, suggesting that this metabolite is readily consumed and produced. In the D3–5 interval, metabolite differentials are not discriminative of embryo quality category at D5 (Roy’s Largest Root = 0.180, F (7, 41) = 1.054, *p* = 0.410, partial η^2^ = 0.153), after controlling for covariates (Figure 3B). Detailed estimates after accounting for covariates are provided in Table 3.

## 3. Discussion

The prevailing approaches employed to assess embryo quality throughout in vitro development exhibit certain limitations. These methods predominantly rely on the expertise of an experienced embryologist for morphological evaluation. Thus, these methodologies are subjective, and there can be inter- and intra-observer variability, which can affect the consistency of embryo grading and impact the selection of the best embryo for transfer. Moreover, morphological assessment is typically performed at specific time points during embryo development, which may not be optimal for evaluating embryo quality [12]. Hence, morphological assessment is not always effective in predicting implantation success. Even high-quality embryos can face challenges in terms of successful implantation or may not culminate in viable pregnancies. This inherent variability can lead to the potential misclassification of certain embryos, thereby contributing to a reduction in the overall success rates of ART treatments. In this sense, the integration of embryo metabolomics with standardized morphological classification can help address some of these limitations by providing a more comprehensive evaluation of embryo quality, including information on potential biomarkers for embryo selection [4].

In this work, we evaluate the metabolic performance of embryos, using ^1^H-NMR spectroscopy, during in vitro development, which were monitored in a time-lapse incubator during a period of 5 days and subject to standardized morphokinetic categorization, according to their development. The application of ^1^H-NMR in the analysis of human IVF culture media comes with both advantages and potential pitfalls. One limitation is the sensitivity of ^1^H-NMR to sample composition. The complex nature of culture media, containing a myriad of compounds, can lead to signal overlap, making it challenging to discern individual metabolites accurately. Additionally, certain metabolites may exist in low concentrations, making their detection and quantification less reliable. Despite these challenges, it is important to highlight that ^1^H-NMR remains a very valuable tool for metabolomic analysis, offering non-destructive, high-throughput, and quantitative insights into the metabolic profiles of biological samples. Researchers must navigate these limitations judiciously, considering them in the context of the specific goals and characteristics of their study, as we did in the present case. Indeed, after standardized morphokinetic categorization of embryos, media were collected to determine those embryos’ metabolic performance. This allows us to search for a possible metabolic biomarker associated with the morphokinetic evaluation.

The definitive classification of embryos was determined through the utilization of Gardner’s blastocyst grading scale [24], with adherence to the guidelines set by the Istanbul Consensus for the timing of observations and the identification of non-viable embryos [13]. Following this classification, they were divided into Good, Lagging, and Bad embryos, as described above. Our goal was to enhance the evaluation of embryo quality by integrating embryo metabolomics with standardized morphokinetic classification, in order to contribute to a more accurate and consistent selection of high-quality embryos with the potential for successful pregnancies. While recognizing that the inclusion of degenerated embryos, non-cleavage embryos, those with abnormal fertilization, and parthenogenetic embryos could yield data with more distinctive differences, we intentionally excluded them to maintain the focus, quality, and consistency of the data. Including embryos with abnormalities or arrested development could introduce confounding variables and detract from the study’s objective, as these embryos are readily identifiable as low-quality with no potential for successful pregnancies.

During the 5-day culture, we chose to correlate the embryo development status with the variation, at D3 and D5, in six specific metabolites that are on the crossroad of major cellular pathways: pyruvate, lactate, alanine, glutamine, acetate, and formate. Indeed, with the exception of glucose (whose consumption remained unaffected across all our experimental conditions—Supplemental Data Figure S1), these metabolites stand as key protagonists within diverse metabolic pathways. Particularly during the intricate process of embryo development, they assume a pivotal role, participating in energy generation, biosynthesis, and intricate signaling cascades [25,26]. For the aforementioned reasons, we hypothesized that one or more of these metabolites may serve as biomarkers for assessing the developmental competence of embryos, providing valuable insights into the underlying metabolic processes that contribute to successful embryogenesis. The identification of metabolites that are sufficiently abundant in the culture media to be easily quantified emphasizes their potential significance as key clinical indicators of the metabolic health and viability of developing embryos. This underscores the rationale for the inclusion of those metabolites in the study and their eventual future relevance in establishing a method that could more or less easily quantify them in the setting of the embryology laboratory.

Pyruvate is the end-product of the energy-yielding process of glycolysis and can be converted to acetyl-CoA, which enters the tricarboxylic acid cycle (TCA) to generate more ATP. Lactate and alanine are produced from pyruvate, particularly when the redox status (NAD+/NADH ratio) is altered. They can be converted back to pyruvate and generate ATP by being driven to the TCA [27]. Acetate is produced during fatty acid metabolism and can be used as an energy source by some cells [28]. Glutamine is an amino acid that is involved in several metabolic pathways, including the synthesis of nucleotides, proteins, and other amino acids, and is also an important source of energy for rapidly dividing cells such as embryos [29]. Formate can be derived from various pathways, including the breakdown of serine, being involved in one-carbon metabolism, which is important for the synthesis of nucleotides and other biomolecules [30]. Research findings have unequivocally shown that human preimplantation embryos actively engage in the consumption and secretion of these metabolites, changing their concentration in the surrounding culture media. This serves as a clear indicator of the embryo’s robust metabolic activity [5,6,12].

Based on our analysis, the adjusted metabolite differentials (for covariates such as the male age, female age, male BMI, female BMI, and ART technique) observed in the first 3 days of embryo culture are discriminative of embryo quality category at the end of D5. The inclusion of covariates related to individual characteristics of the donors of the gametes is crucial to assess the impact of embryo culture medium metabolites in early embryo development. Although ICSI circumvents part of the individual variability, the age and BMI of the donors have been reported to influence the success rate of the ICSI [21,31,32], particularly in European cohorts [33,34]; therefore, we have considered these covariates to add another layer of embryo selection. The major contributors for discriminating Good, Lagging, and Bad embryos were pyruvate, alanine, glutamine, and acetate, after correcting for the confounders. Pyruvate, in the context of embryonic development, serves as an essential energy substrate during the preimplantation stages [27]. This metabolite has been linked to embryo development and increased embryo quality in some species, including cattle and sheep [35]. Although its importance for early embryo development has been evidenced in some studies, the role of pyruvate in human embryonic development is still less clear when other metabolic sources are present [8,27]. In our study, adjusted pyruvate variation in the culture media of 3-day human embryos was quite distinct in Bad embryos from Good and Lagging embryos. While Bad embryos consumed pyruvate, Good and Lagging embryos were able to export and accumulate it in the culture media, suggesting an active metabolism. A similar trend was observed for glutamine, with Good and Lagging embryos being able to export and accumulate this amino acid in the culture media, while Bad embryos consumed it. Glutamine is an important metabolite for embryo development, as it serves as a precursor for various metabolic pathways [9]. It is a non-essential amino acid that can be synthesized by the embryo or taken up from the surrounding culture medium. During early embryonic development, glutamine is predominantly utilized for energy production through the TCA cycle [36,37]. Studies have shown that the addition of glutamine to the culture medium during in vitro embryo development can improve embryo quality and blastocyst formation rates [38]. This suggests that glutamine plays an important role in embryonic metabolism and development [29]. Human embryos have been shown to export glutamine during in vitro development [11]. In vitro studies have shown that the usage of glutamine by embryos increases during the blastocyst stage and that the export of glutamine is associated with increased cell proliferation and blastocyst formation [39], as observed in our experimental work.

Alanine is a non-essential amino acid that also plays an important role in the metabolism of developing embryos. During embryonic development, alanine is involved in the synthesis of proteins, the regulation of glucose metabolism, and the maintenance of cellular redox balance [40]. Studies have shown that alanine is an important nutrient for embryonic growth and development [40]. The availability of alanine can affect the developmental potential of embryos, with lower levels of alanine being associated with decreased embryo quality and lower rates of implantation [39]. In our work, up until D3, Bad embryos produce significantly lower amounts of alanine than Good or Lagging embryos. In fact, during development, the embryo has been shown to produce and export alanine to the surrounding environment, with embryos that produce and export higher levels of alanine having better developmental potential and being linked to higher pregnancy rates [5]. The export of alanine by human embryos is significant because it reflects the metabolic state of the embryo and its ability to maintain energy balance during early development. It has been suggested that the export of alanine may serve as a protective mechanism against stress and nutrient deprivation, allowing the embryo to maintain its viability and developmental potential [41]. Contrastingly, concerning acetate, both Bad and Lagging embryos showed a different trend from that of Good embryos in the variation of this short-chain fatty acid in culture media. While Bad embryos produced higher amounts of acetate in our experimental conditions, Lagging embryos consumed it. Acetate has been shown to be an important energy source for embryo development [6,42]. As the embryo develops and the number of cells increases, the energy demand also increases, and the embryo begins to utilize other energy sources, including acetate [6]. It has been shown that acetate is critical for proper embryo development, as it enhances cell growth and division, regulates gene expression, and improves embryonic quality, particularly for embryos during the preimplantation period [35]. Overall, the higher amounts of acetate observed in the culture media of Bad embryos at the end of D3 should be due to lower utilization of this metabolite due to the poor development of these embryos. The significance of the consumption of acetate by Lagging embryos is unclear, but it may reflect the need to increase the metabolic rates of these cells to enhance the formation of blastocysts.

As a remark, and although no differences were observed in the variation of formate in any of the experimental conditions used, it is of note that our embryos consistently exported significant amounts of this monocarboxylic anion. It is known that mammalian cells generate formate as a byproduct of various metabolic reactions, including the breakdown of certain amino acids and the metabolism of folate. Formate is then either excreted from the cell or used in various biosynthetic pathways, including the synthesis of purines, pyrimidines, and amino acids. In addition, formate can also play a role in regulating cellular redox balance and oxidative stress. For example, formate has been shown to protect cells from oxidative damage by acting as an antioxidant. Overall, the cellular significance of formate in mammalian cells is diverse and multifaceted, with important roles in both metabolism and cellular defense mechanisms [30]. During early embryonic development, formate is produced by the mitochondria and is used as a source of one-carbon units for nucleotide synthesis and methylation reactions [7]. Formate is the main one-carbon donor for de novo purine biosynthesis in the developing embryo. It has been shown that the inhibition of formate metabolism in early embryos can lead to developmental defects and reduced viability [43]. In fact, some studies suggested formate levels as a biomarker for embryonic viability in ART, as the concentration of formate in the culture media can be linked to the metabolic activity of the embryo and in some conditions can be indicative of embryonic viability and quality.

## 4. Materials and Methods

### 4.1. Chemicals

Embryo and gamete culture media and reagents were purchased from Origio^®^ (Malov, Denmark), Nidacon (Molndaln, Sweden), and Vitrolife (Gothenburg, Sweden). All other chemicals were purchased from Sigma-Aldrich (St. Louis, MO, USA) unless stated otherwise.

### 4.2. Patient Selection and Ovarian Stimulation

Twenty-one couples (who underwent a total of 21 cycles) were involved in this study. The treatments of ARTs were conducted at CGR, Porto, Portugal between June 2019 and February 2021. The procedures of the Centre for Reproductive Genetics Alberto Barros are covered by the provisions of the National Medically Assisted Procreation Act (2017) and overseen by the National Council for Medically Assisted Procreation (CNPMA-2018). According to these rules and guidelines, the clinical databases and patient biological material for diagnosis and research may be used without further ethical approval, under strict individual anonymity, and after patient written informed consent. Regarding the use of human samples for laboratory experimentation, the Ethics Committee authorization number is 2021/CE/P02 (P342/2021/CETI), approved on 26 February 2021. The inclusion criteria were couples who were referred to infertility treatment with ICSI technique due to the need to avoid the interference of cumulus cells or spermatozoa during metabolomics quantification. The couple was excluded from the study if they had a testicular biopsy performed (testicular sperm aspiration or testicular sperm extraction) or cryopreserved ejaculated spermatozoa. So, all the ICSI were performed with fresh ejaculated spermatozoa. Semen samples were collected on the day of oocyte retrieval by masturbation. The clinical procedures and laboratory protocols were performed following the CGR standard protocols. Controlled ovarian stimulation was performed using a gonadotropin-releasing hormone antagonist protocol (GnRH-ant). Recombinant follicle-stimulating hormone (rFSH—follitropin alfa: Bemfola^®^, Gedeon Richter, Budapest, Hungary and Gonal F^®^, Merck-Serono, Schiphol-Rijk, The Netherlands; follitropin beta: Puregon^®^, MSD Biotech B.V., Noord-Brabant, The Netherlands), recombinant follicle-stimulating hormone with recombinant luteinizing hormone (rFSH and rLH in a 2:1 ration—Pergoveris^®^, Schiphol-Rijk, Merck-Serono, The Netherlands), menotropin (highly purified human menopausal gonadotrophin, hMG—Menopur^®^, Ferring Pharmaceutical, Madrid, Spain), or corifollitropin alpha (Elonva^®^, N.V. Organon, Oss, The Netherlands) were used to stimulate ovaries with initial doses based on the individual characteristics of patients. Beyond day 6 and when the leading follicle reached 12 mm, treatment was continued with gonadotropin-releasing hormone antagonist ganirelix (Orgalutran^®^, N.V. Organon, Oss, The Netherlands) or cetrorelix acetate (Cetrotide^®^, Merck-Serono, The Netherlands) until final follicular maturation. Stimulation was prolonged until the observation by ultrasound of at least three dominant follicles of 17 mm or greater in diameter or in cases of a low number of growing follicles, at least one. The final oocyte maturation was triggered with 250 μg of recombinant human chorionic gonadotropin (rhCG—choriogonadotropin alfa; Ovitrelle^®^, Merk Serono, Schiphol-Rijk, The Netherlands) or 0.2 mg of GnRH agonist (Triptorelin acetate, Decapeptyl^®^, Ipsen Pharma, Barcelona, Spain) or both. The serum hormone levels of estradiol (E2) and progesterone (P) were evaluated on the day of the trigger. Transvaginal oocyte retrieval was performed 36 h after the final follicular maturation.

In the cases in which embryo transfer was performed, pregnancy was evaluated at two different stages. Women were classified as having a biochemical pregnancy when β-HCG concentration surpassed the value of 20 mIU/mL (positive serum β-HCG levels), 12 days after embryo transfer [24]. All women with negative serum β-HCG levels ([β-HCG] < 20 mIU/mL) were not considered pregnant. Biochemical pregnancies evolved into clinical pregnancies when a fetal heartbeat was detected.

### 4.3. Gamete Collection

After liquefaction, sperm concentration and progressive motility were assessed according to the WHO [44]. The semen preparation standard protocol included a discontinuous density gradient of 90–45% centrifugation (PureSperm^®^, Nidacon, Mölndal, Sweden) followed by a swim-up protocol with fertilization medium (Sequential Fert™, Origio, Ballerup, Denmark). In cases of severe oligoasthenoteratozoospermia, the sperm sample was only washed with a sperm preparation medium (Sperm Preparation Medium™, Origio, Ballerup, Denmark) followed by a swim-up protocol with fertilization medium (Sequential Fert™). The final sperm preparation was incubated at 35 °C with 6% O_2_ at least 30 min before the beginning of the ICSI procedure. After the follicular aspiration, the oocyte–cumulus complexes (OCCs) were isolated and washed in a gamete preparation medium (SynVitro™ Flush, Origio, Ballerup, Denmark). The retrieved OCCs were placed in a 5-well dish with 250 μL of fertilization medium (Sequential Fert™) covered with oil (OVOIL™, Vitrolife, Barcelona, Spain) that had been previously equilibrated overnight (37 °C, 5% CO_2_, and 6% O_2_). OCCs were denuded to evaluate oocyte nuclear maturity. So, 2 h after egg retrieval, cumulus cells were removed using a combination of mechanic and enzymatic (ICSI Cumulase^®^, Origio, Ballerup, Denmark) denudation protocol.

### 4.4. Intracytoplasmic Sperm Injection (ICSI) and Embryo Culture

Four hours after egg retrieval, oocytes were injected following the standard ICSI procedure. Injected oocytes were rinsed and cultivated individually in a 30 μL drop of culture medium (25 μL of culture medium plus 5 μL embryo washed in the same culture medium) (Sequential CleavTM, Origio^®^, Ballerup, Denmark) under 1500 μL of oil (OVOILTM, Vitrolife, Sweden), which was previously equilibrated overnight in an incubator at 37 °C, 5% CO_2_, and 6% O_2_. The culture dish (EmbryoSlide^®^, Vitrolife, Barcelona, Spain) allows an individualized oocyte/embryo culture with no connection between the culture medium of adjacent oocytes/embryos. The culture dish was placed in a time-lapse incubator (EmbryoScope^®^, Vitrolife, Barcelona, Spain) (Day 0) for the following assessment. Normal fertilization was confirmed 16–18 h after ICSI by the presence of two pronuclei and a second polar body (Day 1). Embryo culture was extended until the blastocyst stage (D5). The standard laboratory protocol applied uses a sequential culture media. The embryos were cultured in a cleavage medium (Sequential Cleav™, Origio, Ballerup, Denmark) until D3 and then the culture media were replaced with a blastocyst medium (Sequential Blast™, Origio, Ballerup, Denmark) to support embryo culture until the blastocyst stage (D5). After embryo culture, in some cases, embryos were immediately transferred, while in other cases, embryos were vitrified for subsequent transfer. Thus, we did not evaluate the correlation of our metabolomics results with the success rates in the different types of fertility treatments as it would not be suitable to compare results, because in some treatments the embryo transfer was a fresh embryo transfer and in others, the transfer was deferred.

### 4.5. Embryo Morphology Assessment

The development of each embryo was evaluated from fertilization until D5 in a time-lapse system (Vitrolife, Barcelona, Spain). Routine observation of cleavage embryos (until day 4) mainly includes the number and symmetry of blastomeres, fragmentation percentage and presence of multinucleated blastomeres (day 2/3), and compaction grade (day 4). Cleavage embryos were scored according to four categories (A, B, C, and D), based solely on blastomeres number and fragmentation [45]. The blastocysts were graded according to Gardner Score System [24]. This scoring system uses a number associated with a letter, and this combination allows us to know the blastocyst stage and blastocyst quality. Initially, the classification is based on the degree of expansion and hatching status of blastocysts, and the numeric score from 1 to 6 reflects the blastocyst stage (Table 4).

For blastocysts scored 3–6, the classification system includes the development of the inner cell mass and trophectoderm (Table 4). A blastocyst of good quality was considered as BL3BB or better. The term Lagging was used to characterize those embryos that had acceptable morphological classification, but the embryo development was delayed considering the standardized timing of embryo observations established in the Istanbul Consensus.

### 4.6. Sample Collection/Preparation

As mentioned before, the use of sequential media in an embryo culture implies that until D3 the embryos are cultured in a cleavage medium and afterwards there must be a change in culture medium to support embryo development until D5 (blastocyst stage). So, for each embryo used for the study, there will be two samples of culture medium: a sample of cleavage medium (day 0 until D3 of embryo development) and a sample of blastocyst medium (D3 until D5 of embryo development). Sample collection was carried out by pipetting 25 μL from the 30 μL drop of culture media into microcentrifuge tubes.

Each sample (25 μL) was obtained from the spent culture media of embryos developed from day 0 (after ICSI) until D3 and from D3 until D5. To be used as controls, samples from the same corresponding batch of each medium used in embryo culture and with the same shelf-life were collected and analyzed by ^1^H-NMR spectroscopy as described below and used to evaluate the content of the media at time 0. All samples were centrifuged (1000× *g*; 10 min; 4 °C) to fully separate any residues of oil. Twenty microliters of the bottom layer were carefully collected and stored at −80 °C until metabolomic analysis.

### 4.7. Metabolomic Analysis by ^1^H Nuclear Magnetic Resonance Spectroscopy

1D ^1^H-NMR spectroscopy was applied to identify and quantify metabolites in the embryo culture media. NMR spectra were acquired using a 600 MHz VNMRS spectrometer (Varian, Inc. Palo Alto, CA, USA) equipped with a 3 mm indirect detection probe. About 140 µL of deuterium oxide and 40 µL of sodium fumarate solution (final concentration of 2 mM) were added to 20 µL of each embryo culture media, and the final volume of 200 µL was transferred into a 3 mm NMR tube for analysis. Sodium fumarate was used as an internal reference (6.50 ppm) to quantify the following metabolites (multiplicity, chemical shift (ppm)): alanine (doublet, 1.46), acetate (singlet, 1.90), pyruvate (singlet, 2.35), glutamine (multiplet, 2.45), lactate (quartet, 4.09), and formate (singlet, 8.44). NMR spectra were processed by applying exponential line broadening (0.2 Hz), baseline correction, and manual phasing, and the areas of the resonances were quantified using the NUTSpro^TM^ NMR Software (2D Professional Version-20070315, Acorn, Fremont, CA, USA). Blank control experiments were performed previously in which we replicated every step of the incubation except that there were no embryos being cultivated. With this procedure, we were able to assess the impact of the protein content (with HSA ≤ 0.05%) on the NMR spectra acquisition, ensuring that there were no signals generated that overshadowed the metabolites of interest. We were also able to perfect the separation of the oil from the spent culture (in order to obtain clear NMR spectra without disturbing overlapping or nearby signals derived from the oil), and we analyzed the impact of this incubation on the content of the medium in water-soluble metabolites, as the ones we evaluated in the experiment. We saw no alteration in the content of these metabolites, in line with the results described by a previous study [46]. Moreover, fresh medium samples were collected for each distinct batch and within the same medium batch if different shelf times were used (21 control samples for D3 and 21 samples for D5). Subsequently, they were analyzed by ^1^H-NMR together with correspondent spent media (same batch plus same shelf time) and used to calculate the initial metabolite content and subsequently the metabolite production or consumption.

### 4.8. Statistical Analysis

Univariate statistics were used to compare embryo culture medium metabolite differential in days 0–3 (D0–3) and 3–5 (D3–5) between embryos grouped by their assessed quality at D5 (Good vs. Lagging vs. Bad). Data are presented as mean ± standard deviation (S.D.). Embryo quality was assessed by the same trained and experienced embryologist. Normality and heteroscedasticity tests were performed, and univariate ANOVA was conducted on data collected concerning variation in selected metabolites identified in the embryo culture medium. Pairwise comparisons between groups were corrected for multiple hypotheses testing by Tukey’s post hoc test. Multivariate analysis of metabolite consumption/production was performed using a linear model integrating corrections for confounding factors. Metabolite differentials in days 0–3 (D0–3) and 3–5 (D3–5) were compared between embryos grouped by their assessed quality at D5 (Good vs. Lagging vs. Bad) using multivariate analysis of covariance (MANCOVA), after controlling for covariates (male age, female age, male BMI, female BMI). These covariates were chosen based on multiple studies correlating maternal age [14,16] and BMI [18,19], and paternal age [20] and BMI [21,23] with changes in early embryo metabolism and viability [17,22]. The assumptions of equality of the variance–covariance matrices were evaluated using Levene’s test of equality of error variances and Box’s test of equality of covariance matrices (Box’s M). The discriminant value of the independent variable (embryo quality at D5) considering the dependent variables (metabolite differential) was evaluated according to Roy’s Largest Root criterion. This method was chosen as it is the most sensitive when a limited number of dependent variables is responsible for the differences between groups of the independent variable [47]. The impact of each metabolite differential on embryo quality at D5 was assessed using a univariate F-test, after adjusting for the covariates. Pairwise comparisons between groups were corrected for multiple hypotheses testing by Šidak’s method. All statistical tests of hypotheses for all statistical methods were performed at the α = 0.05 level using SPSS Statistics 28.0.0 for Windows 64-bit (IBM, Armonk, NY, USA).

## 5. Conclusions

The goal of integrating metabolomics with standardized embryonic viability assessment could be crucial for non-invasive, real-time monitoring of embryo metabolic states during culture, offering valuable information on viability and quality. Current methods, such as morphological assessment and morphokinetic analyses, have limitations in predicting implantation success, making metabolomics an essential addition. Furthermore, this integration can lead to identifying biomarkers associated with embryonic viability and quality, potentially improving embryo selection for transfer and enhancing the chances of successful pregnancies. Still, our strategy does not address metabolic differences between inner cell mass (ICM) and trophectoderm, noted in previous reports showing distinct metabolic rates between these cells [48,49]. Moreover, the metabolic activity of ICM cells was suggested to play a role in embryo development [49,50]. Yet, the methods applied in these studies destroy or compromise the viability of the embryos. Existing methods compromising embryo viability highlight a need for non-destructive techniques. Alegre et al. proposed Raman spectroscopy, estimating the oxidant content of the culture medium, but its external variability and lower specificity compared to NMR or MS spectroscopy raise concerns [4]. Hence, NMR emerges as a potential compromise, as demonstrated in recent studies of early-stage mammalian embryos [51]. Despite evidence supporting metabolic screening’s benefits for nearly a decade, routine implementation in ART clinics is lacking [52,53].

**Figure 4:**
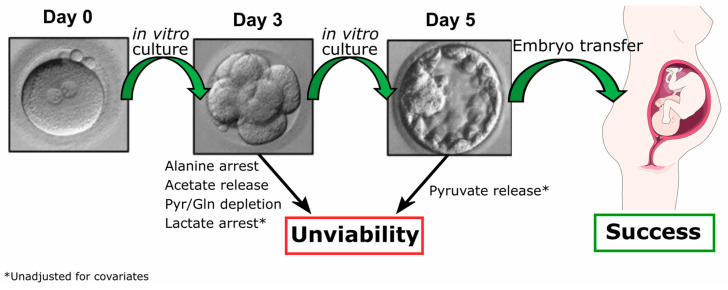
Metabolite variation in human embryo culture medium is a predictor of successful pregnancy in ARTs. Embryo metabolism is indirectly assessed by comparison of metabolite concentration in culture medium before and after incubation. We observed that Poor-quality embryos at post-fertilization day 5 displayed already anomalies in alanine, acetate, pyruvate, and glutamine metabolism, especially compared to embryos classified as Lagging at day 5. These differences were found after adjusting for covariates (male age, female age, male BMI, female BMI). Considering unadjusted variables, lactate is another potential success predictor for ARTs at day 3. On day 5, considering unadjusted variables, net-zero pyruvate is a feature of good-quality embryos.

Overall, our results support that assessment of the exometabolome of in vitro cultured human embryos up to D3 can be discriminative of embryo quality category at D5. Notably, the metabolite profile at D3 is particularly selective between Lagging and Bad-quality embryos, whereas the metabolite profile of Good embryos is not different from Lagging embryos (except for alanine) at this stage. However, at D5, lactate is a potential predictor between Good and Lagging embryos. Therefore, the corrected variations (for interfering covariates, such as the male age, female age, male BMI, and female BMI) of metabolites such as alanine, pyruvate, acetate, and glutamine support that the integration of metabolomics with the standardized assessment of embryonic viability and quality has the potential to improve the efficiency and effectiveness of ARTs (Figure 4), leading to better pregnancy rates and improved clinical outcomes.

While the number of samples used in this study is low and may hamper some more definite conclusions, these findings suggest a potential advancement in refining embryo selection procedures. Incorporating metabolic assessments into protocols could improve ART outcomes by enhancing grading precision and potentially boosting implantation success. This study contributes to ongoing efforts to optimize ART procedures, offering a more comprehensive approach to embryo quality assessment beyond relying solely on morphological evaluation. The potential application of metabolic assessments as additional tools in embryo selection may represent progress in reproductive medicine.

## Figures and Tables

**Table 1:** Characterization of the studied population and fertilization cycles.

Baseline Characteristics of Studied Population		Patient Cycle Characteristics	
Number of couples	21	Fertilization technique (%)	
Number of cycles	21	ICSI	100 (21/21)
Female age (years)	35.05 ± 6.59	Stimulation duration (days)	9.29 ± 1.38
Female body mass index (kg/m^2^)	23.59 ± 3.49	FSH total dose (IU)	2827 ± 1245
Male age (years)	40.71 ± 5.49	Serum E2 on trigger day (pg/mL)	2481 ± 1486
Male body mass index (kg/m^2^)	24.84 ± 5.82	Serum Pr on trigger day (ng/mL)	0.95 ± 0.46
Infertility duration (months)	49.86 ± 32.29	Oocytes retrieved (n)	8.48 ± 2.66
Previous IVF attempts (%)		Metaphase II oocytes (n)	7.43 ± 2.31
0	42.9 (9/21)	Maturation rate (%)	85.2 ± 14.4
1	23.8 (5/21)	Fertilized oocytes (n)	6.33 ± 2.46
2	19.0 (4/21)	Fertilization rate (%)	85.20 ± 14.38
≥3	14.3 (3/21)	Embryo stage	
Types of infertility (%)		Day 3 embryos (n)	6.14 ± 2.5
Primary infertility	66.7 (14/21)	Day 5 embryos (n)	4.95 ± 2.41
Secondary infertility	33.3 (7/21)	Cleavage rate (%)	96.7 ± 7.1
Anti-Müllerian hormone (ng/mL)	4.02 ± 4.34	Embryo utilization rate (%)	53.6 ± 21.5
Total number of analyzed embryos	55	Embryo transfers (%)	92.9 (13/14)
Good	26	1 embryo transferred	69.2 (9/13)
Lagging	19	2 embryos transferred	30.8 (4/13)
Bad	10	Freeze all cycles	7.1 (1/14)
		Biochemical pregnancy (%)	61.5 (8/13)
		Clinical pregnancy (%)	61.5 (8/13)

Data are shown as the mean ± standard deviation or %. Legend: E2, estradiol; FSH, follicle-stimulating hormone; ICSI, intracytoplasmic sperm injection; Pr, progesterone.

**Table 2:** Metabolite variation in embryo culture media. Metabolite variation observed on the first 3 days (D0–3) and from day 3 to day 5 (D3–5) in culture media of human embryos grouped according to their quality (Good, Lagging, and Bad).

Metabolite (nmol/Embryo)	Metabolite Variation in Embryo Culture Media					
	Day 0 to 3			Day 3 to 5		
	Good	Lagging	Bad	Good	Lagging	Bad
Alanine	2.63 ± 1.68	3.34 ± 1.65	1.42 ± 0.57 *^,#^	4.92 ± 2.83	5.05 ± 2.62	5.27 ± 2.70
Acetate	1.59 ± 5.23	−0.94 ± 4.98	3.11 ± 3.66 ^#^	−1.43 ± 4.44	1.04 ± 5.78	−1.24 ± 4.16
Pyruvate	0.10 ± 1.23	0.81 ± 0.94 *	0.35 ± 0.42	0.23 ± 0.67	0.78 ± 0.38	0.33 ± 0.57
Glutamine	3.16 ± 4.94	5.24 ± 2.69	0.80 ± 4.66 ^#^	3.10 ± 4.47	4.48 ± 5.65	4.90 ± 3.42
Lactate	11.55 ± 15.05	19.44 ± 9.05 *	7.49 ± 3.99 ^#^	16.60 ± 13.27	19.03 ± 10.39	15.55 ± 10.99
Formate	6.00 ± 1.18	5.82 ± 1.06	5.54 ± 0.94	5.85 ± 1.51	6.33 ± 1.21	6.43 ± 1.23

Data are shown as mean ± SD. Positive values represent release to the extracellular medium, while negative values mean consumption by the embryo. All *p* values < 0.05 were considered statistically significant (* vs. Good embryos, ^#^ vs. Lagging embryos).

**Table 3:** Adjusted metabolite variation in embryo culture media. Metabolite variation observed on the first 3 days (D0–3) and from day 3 to day 5 (D3–5) in culture media of human embryos grouped according to their quality (Good, Lagging, and Bad).

Metabolite (nmol/Embryo)	Adjusted Metabolite Variation in Embryo Culture Media ^a^					
	Day 0 to 3			Day 3 to 5		
	Good	Lagging	Bad	Good	Lagging	Bad
Alanine	2.78 ± 1.45	3.25 ± 1.44	1.11 ± 1.48 *^,#^	5.15 ± 3.33	4.75 ± 3.32	6.22 ± 3.40
Acetate	1.15 ± 4.20	−0.95 ± 4.19	4.50 ± 4.29 ^#^	0.72 ± 5.88	0.86 ± 5.86	−2.67 ± 6.00
Pyruvate	0.25 ± 0.97	0.61 ± 0.97	−0.45 ± 0.99 ^#^	0.28 ± 0.61	0.66 ± 0.61	0.29 ± 0.62
Glutamine	3.59 ± 4.05	3.98 ± 4.03	−0.22 ± 4.14 ^#^	3.27 ± 5.18	4.57 ± 5.15	6.22 ± 5.28
Lactate	13.07 ± 12.13	14.69 ± 12.08 *	4.09 ± 12.38 ^#^	18.26 ± 17.04	15.00 ± 16.96	17.21 ± 17.38
Formate	5.10 ± 2.36	4.81 ± 2.34	5.39 ± 2.41	5.37 ± 2.54	5.26 ± 2.53	5.90 ± 2.59

Data are shown as mean ± SD. Positive values represent release to the extracellular medium, while negative values mean consumption by the embryo. ^a^ Covariates: Female Age = 34.52, Male Age = 39.43, Female BMI = 23.611934, Male BMI = 26.344065, technique = 2.80. All *p* values < 0.05 were considered statistically significant (* vs. Good embryos, ^#^ vs. Lagging embryos).

**Table 4:** Classification of blastocysts, inner cell mass, and trophectoderm. Degree of expansion and hatching status of blastocysts. Gardner blastocysts grade system was assessed as indicated in the table. Inner cell mass and trophectoderm classification for onward full blastocyst stage (BL3 to BL6).

**Blastocyst Classification**	**Stage**	**Definition**	**Examples**	**Inner Cell Mass Classification**	**Definition**	**Examples**
**BL1**	Early blastocyst	The blastocoel being less than half the volume of the embryo	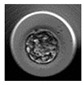	**A**	Tightly packed with many cells	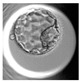
**BL2**	Blastocyst	The blastocoel being greater than half the volume of the embryo	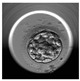	**B**	Loosely grouped with several cells	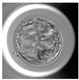
**BL3**	Full blastocyst	The blastocoel completely filling the embryo	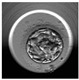	**C**	Very few cells	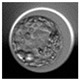
**BL4**	Expanded blastocyst	The blastocoel volume now being larger than that of the early embryo and the zona pellucida starting to thin	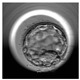	**Trophectoderm Classification**	**Definition**	**Examples**
				**A**	Many cells forming a cohesive epithelium	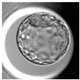
**BL5**	Hatching blastocyst	The trophectoderm starting to herniate though the zona pellucida	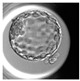	**B**	Few cells forming a loose epithelium	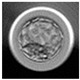
**BL6**	Hatched blastocyst	The blastocyst having completely escaped from the zona pellucida	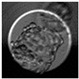	**C**	Very few large cells	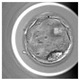

## Data Availability

Data is contained within the article and Supplementary Materials.

## Supplementary Materials

The following supporting information can be downloaded at: https://www.mdpi.com/article/10.3390/ijms25010491/s1.
