# Supplementary material for: Metabolomics Integration in Assisted Reproductive Technologies for Enhanced Embryo Selection beyond Morphokinetic Analysis
Source: Int J Mol Sci. 2023 Dec 29;25(1):491. doi: 10.3390/ijms25010491 (PMC10778973; doi:10.3390/ijms25010491)
Supplement: Supplementary file 1 [file ijms-25-00491-s001.zip › ijms-2725386-supplementary.pdf]

SUPPLEMENTAL DATA

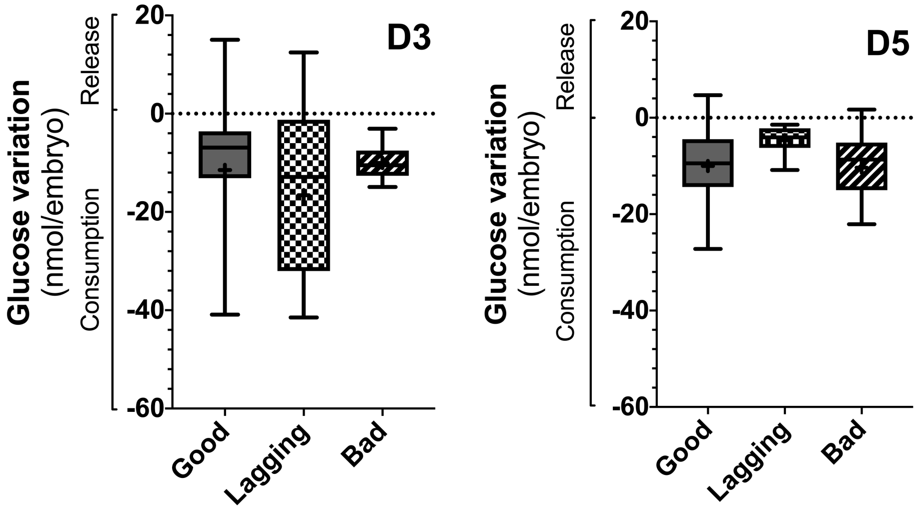

Supplemental Figure S1. Glucose variation in culture media of human embryos between day 3 and day 0 (D3) or day 5 day 3 (D5) grouped according to their quality at day 5 (Good vs. Lagging vs. Bad). The figure shows the consumption of glucose quantified in the embryo culture media at the end of day 3 or day (n=55). Data are presented as Mean ± Standard Deviation (S.D.). Normality and heteroscedasticity tests were performed, and Univariate ANOVA was conducted on the selected metabolites identified in the embryo culture medium. Pairwise comparisons between groups were corrected for multiple hypotheses testing by Tukey's post hoc test. All p values < 0.05 were considered statistically significant.
